# Cardiovascular Responses to Chemical Stimulation of the Hypothalamic Arcuate Nucleus in the Rat: Role of the Hypothalamic Paraventricular Nucleus

**DOI:** 10.1371/journal.pone.0045180

**Published:** 2012-09-17

**Authors:** Tetsuya Kawabe, Kazumi Kawabe, Hreday N. Sapru

**Affiliations:** Department of Neurological Surgery, University of Medicine and Dentistry of New Jersey- New Jersey Medical School, Newark, New Jersey, United States of America; Hosptial Infantil Universitario Niño Jesús, CIBEROBN, Spain

## Abstract

The mechanism of cardiovascular responses to chemical stimulation of the hypothalamic arcuate nucleus (ARCN) was studied in urethane-anesthetized adult male Wistar rats. At the baseline mean arterial pressure (BLMAP) close to normal, ARCN stimulation elicited decreases in MAP and sympathetic nerve activity (SNA). The decreases in MAP elicited by ARCN stimulation were attenuated by either gamma-aminobutyric acid (GABA), neuropeptide Y (NPY), or beta-endorphin receptor blockade in the ipsilateral hypothalamic paraventricular nucleus (PVN). Combined blockade of GABA-A, NPY1 and opioid receptors in the ipsilateral PVN converted the decreases in MAP and SNA to increases in these variables. Conversion of inhibitory effects on the MAP and SNA to excitatory effects following ARCN stimulation was also observed when the BLMAP was decreased to below normal levels by an infusion of sodium nitroprusside. The pressor and tachycardic responses to ARCN stimulation at below normal BLMAP were attenuated by blockade of melanocortin 3/4 (MC3/4) receptors in the ipsilateral PVN. Unilateral blockade of GABA-A receptors in the ARCN increased the BLMAP and heart rate (HR) revealing tonic inhibition of the excitatory neurons in the ARCN. ARCN stimulation elicited tachycardia regardless of the level of BLMAP. ARCN neurons projecting to the PVN were immunoreactive for glutamic acid decarboxylase 67 (GAD67), NPY, and beta-endorphin. These results indicated that: 1) at normal BLMAP, decreases in MAP and SNA induced by ARCN stimulation were mediated via GABA-A, NPY1 and opioid receptors in the PVN, 2) lowering of BLMAP converted decreases in MAP following ARCN stimulation to increases in MAP, and 3) at below normal BLMAP, increases in MAP and HR induced by ARCN stimulation were mediated via MC3/4 receptors in the PVN. These results provide a base for future studies to explore the role of ARCN in cardiovascular diseases.

## Introduction

The hypothalamic arcuate nucleus (ARCN) is located bilaterally adjacent to the floor of the third ventricle. Much of our information regarding the chemical nature of ARCN neurons has been accumulated from the investigations on feeding behavior and energy homeostasis [1]–[5]. Based on these reports, the existing information regarding the chemical nature of major populations of ARCN neurons can be summarized as follows. The ARCN contains the highest number of neuropeptide Y (NPY) expressing neurons [6], [7]. More than 90% of NPY containing neurons co-express agouti-related peptide (AgRP) [2]. NPY/AgRP neurons are located in the ventromedial portion of the ARCN. Gamma-aminobutyric acid (GABA) is present in many NPY/AgRP neurons in the ARCN [8], [9]. These neurons serve orexigenic functions (increase in feeding and decrease in energy expenditure including decrease in sympathetic nerve activity [SNA]). Anorexigenic functions (decrease in feeding and increase in energy expenditure including increase in SNA) are served by neurons containing proopiomelanocortin (POMC) and cocaine and amphetamine-regulated transcript (CART) (POMC/CART neurons) which are located in the ventrolateral ARCN. POMC is the precursor of other peptides such as alpha-melanocyte stimulating hormone (alpha-MSH), beta-endorphin and adrenocorticotropin (ACTH) [10]. Thus, POMC/CART neurons in the ARCN also contain alpha-MSH, beta-endorphin and ACTH [11], [12]. There are two separate subpopulations of POMC/CART neurons in the ARCN: one containing GABA and other containing glutamate [13], [14]. Both NPY/AgRP and POMC/CART neurons in the ARCN project to the hypothalamic paraventricular nucleus (PVN) [5]. Stimulation of NPY/AgRP neurons in the ARCN results in the release of NPY/AgRP in the PVN promoting food intake and decrease in energy expenditure and SNA. Stimulation of POMC/CART neurons in the ARCN results in the release of one of the excitatory neurotransmitters (alpha-MSH) in the PVN; alpha-MSH activates melanocortin receptors (MC3/4 receptors) in the PVN and inhibits food intake and increases energy expenditure and SNA. NPY/AgRP and POMC/CART neurons are regulated by peripheral hormones (e.g., leptin and insulin) as well as nutrients (e.g., glucose, amino acids and fatty acids). Leptin and insulin inhibit NPY/AgRP and stimulate POMC/CART neurons [4], [15], [16].

There is a general consensus that the PVN plays an important role in the central cardiovascular regulation [17]. For example, chemical stimulation or disinhibition of the PVN has been reported to elicit increases in blood pressure (BP) and SNA [18]–[21]. The PVN is known to project to the rostral ventrolateral medullary pressor area (RVLM) and the intermediolateral cell column of the spinal cord (IML) [22]. Disinhibition and chemical stimulation of the PVN has been reported to excite spinally projecting RVLM neurons [19]–[21], [23]–[26]. Furthermore, excitation of spinally projecting RVLM neurons in response to the chemical stimulation of the PVN was mediated via glutamate in the RVLM [27].

There are very few studies in which the ARCN has been stimulated chemically to evaluate its role in cardiovascular regulation [16], [17], [28], [29]. In view of the complexity of the chemical nature of different populations of ARCN neurons, diverse cardiovascular responses are expected following the chemical stimulation of the ARCN. We have previously reported that stimulation of ARCN by microinjections of N-methyl-D-aspartic acid (NMDA) elicits increases in BP, heart rate (HR) and SNA [29]. As mentioned earlier, presence of GABA, beta-endorphin and NPY containing neurons has been reported in the ARCN [8], [9], [11]–[14]. GABA, beta-endorphin and NPY have been reported to exert inhibitory effects on neurons [30]–[32]. One of the targets of the ARCN neurons is the PVN which plays an important role in regulating cardiovascular function [5], [17], [33]–[35]. Based on this information, it was hypothesized that decreases in BP and SNA can be elicited by the chemical stimulation of the ARCN and these responses may be mediated via the PVN. This hypothesis was tested in the present investigation.

## Materials and Methods

### Ethics Statement

The experiments were performed according to the NIH guide for “The Care and Use of Laboratory Animals, 7th Edition, 1996” and with the approval of the Institutional Animal Care and Use Committee (IACUC) of UMDNJ-New Jersey Medical School, Newark, NJ, USA (Approval #: 11140D0215). Every effort was made to prevent the suffering of the animals and minimize their distress.

### General Procedures

Experiments were done in adult male Wistar rats (Charles River Laboratories, Wilmington, MA, USA) weighing 300–360 g. All animals were housed under controlled conditions with a 12-h light/dark cycle. Food and water were available to the animals ad libitum.

The general procedures have been described in detail elsewhere [36]. Briefly, the rats were anesthetized with inhalation of isoflurane (2–3% in 100% oxygen). A tracheostomy was performed and the rats were artificially ventilated using a rodent ventilator (model 683; Harvard Apparatus, Holliston, MA, USA). The tidal volume and frequency were adjusted on the ventilator to maintain the end tidal CO_2_ at 3.5–4.5%. One of the femoral arteries was cannulated for monitoring BP. Mean arterial pressure (MAP) and HR were derived electronically from BP waves. One of the femoral veins was cannulated and urethane (1.2–1.4 g/kg) was injected intravenously in 8–9 aliquots at 2-min intervals (total volume of the anesthetic solution was 0.4–0.45 ml injected over a period of about 16–18 min). Isoflurane inhalation was terminated as soon as urethane administration was completed. Absence of a BP response and/or withdrawal of the limb in response to pinching of a hind paw indicated that the rats were properly anesthetized. Rectal temperature was maintained at 37±0.5°C using a temperature controller (model TCAT-2AC, Physitemp Instruments, Clifton, NJ, USA). All of the tracings were stored on a computer hard drive using a data acquisition system obtained from Cambridge Electronic Design Ltd (CED; Cambridge, UK). At the end of the experiment, the rats were deeply anesthetized with a high dose of urethane (2 g/kg, i.v.), a pneumothorax was produced by an incision in one of the intercostal muscles and cessation of heart beat indicated that euthanasia was complete.

### Microinjection Technique

The details of this technique are described elsewhere [18]. Briefly, the rats were placed in a prone position in a stereotaxic instrument (David Kopf Instruments, Tajunga, CA, USA) with bite bar 3.3 mm below the interaural line. The bregma was visually identified and a small hole was drilled in the parietal bone. Multi-barreled glass-micropipettes (tip size 20–40 µm) were used for microinjections. For microinjection into the ARCN, the micropipettes were inserted into the brain perpendicularly. The coordinates for microinjections into the ARCN were: 1.9–4.1 mm caudal to the bregma, 0.2–0.4 mm lateral to the midline, and 9.6–9.9 mm deep from the dura. For microinjections into the ARCN and the PVN in the same experiment, the micropipettes were inserted into the PVN perpendicularly and at a 10°-angle rostrally into the ARCN; in these experiments, the middle or caudal region of ARCN was stimulated by the microinjections of NMDA. The coordinates for the microinjections into the ARCN were: 4.0–5.4 mm caudal to the bregma, 0.2–0.4 mm lateral to the midline, and 9.7–10.0 mm deep from the dura and the coordinates for microinjections into the PVN were: 1.6–1.9 mm caudal to the bregma, 0.3–0.5 mm lateral to the midline and 7.7–8.0 mm deep from the dura. The ARCN and/or PVN were always stimulated by unilateral microinjections of NMDA (10 mM). The duration of microinjection was 10 sec. Controls for microinjections consisted of artificial cerebrospinal fluid (aCSF, pH 7.4). The rats were paralyzed with intravenous administration of pancuronium bromide (initial bolus injection of 1.2 mg/kg followed by 0.6 mg/kg bolus injections every 40 min) in order to avoid cardiovascular effects secondary to respiratory changes following ARCN stimulation. In order to investigate the site specificity of the responses elicited by ARCN stimulation, NMDA was microinjected into the hypothalamic dorsomedial (DMN) and ventromedial (VMN) nuclei and the third ventricle. The coordinates for microinjections into the DMN were: 3.0–3.2 mm caudal to the bregma, 0.5–0.6 mm lateral to the midline and 8.5–8.9 mm deep from the dura. The coordinates for microinjections into the VMN were: 2.9–3.0 mm caudal to the bregma, 0.5–0.6 mm lateral to the midline and 9.4–9.6 mm deep from the dura. The coordinates for microinjections into the third ventricle were: 3.7–3.8 mm caudal to the bregma, 0.0 mm lateral to the midline and 9.5–9.8 mm deep from the dura. The volumes of all microinjections into the ARCN and PVN were 20 and 30 nl, respectively.

### Greater Splanchnic Nerve Recording

The details of recording from the greater splanchnic nerve (GSN) are mentioned elsewhere [36]. Briefly, the GSN was sectioned at its junction with the celiac ganglion, a small segment was desheathed and its activity was recorded using a bipolar silver wire hook electrode. The activity of whole GSN (GSNA) was amplified (×10,000–20,000), filtered (100–5000 Hz), digitized and stored on a computer hard drive. The digitized signals were full-wave rectified and integrated over consecutive 1 sec intervals using Spike 2 program (CED, UK). At the end of the experiment, the nerve was sectioned centrally and the remaining activity was considered to be the noise level which was subtracted from the GSNA amplitude.

### Retrograde Tracing of ARCN Projections and Immunohistochemistry

The surgery for the tracing studies was done under aseptic conditions. The rats were anesthetized with pentobarbital sodium (50 mg/kg, i.p.), fixed in a prone position in a stereotaxic instrument and green retrobeads IX (original undiluted solution supplied by Lumafluor Inc.) were microinjected (30 nl) into the PVN. Absorbable gelatin sponge (Surgifoam, Ethicon Inc., Somerville, NJ, USA) was placed on the cerebral surface and the skin over the wound was sutured. The rats were kept alive for a total of 7 days. An antibiotic (cefazolin, 30 mg/kg) and an analgesic (buprenorphine, 0.05 mg/kg) were administered subcutaneously twice a day for 3 days. On the fifth day after the surgery, the rats were again anesthetized with pentobarbital and colchicine (120 µg, 10 µl) was microinjected into the lateral ventricle unilaterally. The coordinates for microinjections into the lateral ventricle were: 0.8–0.9 mm caudal to the bregma, 1.7–1.8 mm lateral to the midline, and 3.8–4.0 mm deep from the dura. On the seventh day, the animals were then deeply anesthetized with pentobarbital (80 mg/kg, i.p.), perfused first with heparinized normal saline which was followed by 2% paraformaldehyde solution containing 0.2% picric acid. The brains were removed and placed in 2% paraformaldehyde containing 0.2% picric acid for 48 hrs. On completion of the fixation procedure, one side of the brain surface was marked by a shallow cut and serial sections of the hypothalamic area were cut (40 µm) in a vibratome (1000 Plus Sectioning System, The Vibratome Company, St. Louis, MO, USA). The microinjection site of green retrobeads IX (Amax = 460 nm, Emax = 505 nm) and the retrogradely-labeled cells were visualized under a microscope (model AX70, Olympus Provis, Middlebush, NJ, USA). The sections were photographed (Neurolucida software, version 7.5, MicroBrightField Inc., Williston, VT, USA) and compared with a standard atlas [37].

After the retrograde labeling of ARCN neurons was confirmed, some sections containing the ARCN were used for immunostaining of the following peptides: NPY, beta-endorphin, and glutamic acid decarboxylase 67 (GAD67). The sections were rinsed (rinsing of sections was always done 3 times, 10 min each) with 0.1 M phosphate buffered saline (PBS) and blocked for 60 min at room temperature with 10% normal goat serum (NGS) in 0.1 M PBS containing 0.3% Triton X-100 (TPBS). For NPY staining, the sections were then incubated for 24 hours at 4°C with rabbit anti-NPY antibody (1∶200; Phoenix Pharmaceuticals Inc; Burlingame, CA, USA; diluted with TPBS containing 3% NGS). After rinsing with PBS, the sections were incubated for 2 hours at 4°C with Cy3-goat anti-rabbit IgG (1∶200, Amax = 550 nm, Emax = 570 nm, Jackson Immuno-Research Laboratories Inc., West Grove, PA, USA; diluted with PBS containing 3% NGS). For beta-endorphin staining, the same procedures were carried out except that rabbit anti-beta-endorphin (1∶400; Phoenix Pharmaceuticals Inc.) was used instead of anti-NPY antibody. For GAD67 staining, sections were immersed in 1% hydrogen peroxide for 10 min and then rinsed with distilled water before incubation with NGS in PBS. The sections were then incubated with mouse anti-GAD67 monoclonal antibody (1∶1000; Millipore Corp., Temecula, CA, USA; diluted with TPBS containing 3% NGS) for 24 hours at 4°C. After rinsing with PBS, the sections were incubated with Cy3-goat anti-mouse IgG (1∶200; Jackson Immuno-Research Laboratories, Inc; diluted with PBS containing 3% NGS) for 2 hrs at 4°C. In each series of immunohistochemistry experiments, after the completion of incubation with the primary and secondary antibodies, the sections were rinsed in PBS, mounted on subbed slides, covered with Citifluor mountant medium (Ted Pella Inc., Redding, CA, USA) and coverslipped. The images of the sections were captured, 1 µm apart, by laser scanning confocal microscopy (AIR confocal microscope, Nikon Instruments Inc., Melville, NY, USA).

### Histological Identification of Microinjection Sites

At the end of the experiment, diluted green retrobeads IX (1∶50) were microinjected into the ARCN and the PVN as a marker to confirm microinjection sites. The animals were perfused and fixed with 2% paraformaldehyde and serial sections of the hypothalamus were cut (40 µm) in a vibratome and mounted on slides. The microinjection sites were identified under a microscope as mentioned above. The sections were photographed and compared with a standard atlas [37].

### Drugs and Chemicals

The following drugs and chemicals were used: NMDA, gabazine (GABA-A receptor antagonist), naloxone hydrochloride (a competitive antagonist at mu, kappa and delta opioid receptors), BMS193885 (NPY1 receptor antagonist), SF11 (NPY2 receptor antagonist), green retrobeads IX, l-phenylephrine hydrochloride, pancuronium bromide, isoflurane, urethane, pentobarbital sodium, cefazolin, buprenorphine hydrochloride, sodium nitroprusside (SNP), AgRP, SHU9119 (Ac-Nle-cyclo(-Asp-His-D-2-Nal-Arg-Trp-Lys)-NH2; MC3/4 receptor antagonist), L-glutamate monosodium (L-Glu) and D-AP7 (D-(-)-2-Amino-7-phosphonoheptanoic acid; NMDA receptor antagonist). All of the solutions for the microinjections were freshly prepared in aCSF except BMS193885 which was dissolved in distilled water (pH 7.4). The composition of aCSF (pH 7.4) was as follows: NaCl (128 mM), KCl (3 mM), CaCl_2_ (1.2 mM), MgCl_2_ (0.8 mM), dextrose (3.4 mM) and HEPES (5 mM). Where applicable, the concentration of drugs refers to their salts. The vendors for different drugs and chemicals were as follows: BMS193885, SF11, SHU9119 and D-AP7 (Tocris Bioscience, Ellisville, MO, USA), AgRP (Phoenix Pharmaceuticals Inc.), isoflurane (Baxter Pharmaceutical Products, Deerfield, IL, USA), pentobarbital (Ovation Pharmaceuticals Inc., Deerfield, IL, USA), cefazolin (West-ward Pharmaceutical Corporation, Eatontown, NJ, USA), buprenorphine (Hospira Inc., Lake Forest, IL, USA), and green retrobeads IX (Lumafluor Inc., Durham, NC, USA). All other drugs and chemicals were obtained from Sigma Chemicals (St. Louis, MO, USA).

### Statistical Analyses

The means and standard error of the means (S.E.M.) were calculated for maximum changes in MAP and HR in response to microinjections of different drugs. One-way analysis of variance (ANOVA) followed by Tukey-Kramer multiple test was used for determination of concentration-response and tachyphylaxis of NMDA responses in the ARCN and for comparison of NMDA responses from different regions of the ARCN. Student’s paired t-test was used for comparison of the following responses: increases in MAP and HR induced by the microinjections of NMDA into the ARCN before and after the microinjections of BMS193885, naloxone and/or gabazine into the PVN or ARCN. For analyses of the GSNA, the integrated signals obtained just before the microinjections of NMDA into the ARCN were averaged over a period of 60 sec. When the responses to these treatments were maximal, the integrated signals were averaged over a period of 60–90 sec. The percentage changes in SNA elicited by these treatments were calculated and compared by using Student’s paired t-test. In all cases, the differences were considered significant at P<0.05.

## Results

Baseline values for MAP and HR in urethane-anesthetized rats were 99.7±1.1 mmHg and 439.7±3.8 beats/min (bpm), respectively (n = 98).

### 1. Depressor Responses to Microinjections of NMDA in the ARCN

Microinjections of NMDA (2.5, 5, and 10 mM) into the ARCN elicited the decreases in MAP and increases in HR (Table. 1). The responses to 5 and 10 mM NMDA were not statistically different; 10 mM concentration of NMDA was selected for further studies in other groups of rats. The onset, peak and duration of the responses elicited by 10 mM concentration of NMDA were 5–15 sec, 1–3 min, and 10–15 min, respectively. Microinjections (20 nl) of L-Glu (100 and 500 mM, n = 4 each) also elicited small decreases in MAP (2.5±0.6 and 4.0±0.4 mmHg, respectively) and increases in HR (11.5±3.7 and 15.0±2.2 bpm, respectively). The responses to both concentrations of L-Glu (100 and 500 mM) were significantly smaller (P<0.05) when compared with those elicited by NMDA (10 mM). Therefore, NMDA (10 mM) was selected for the chemical stimulation of the ARCN in all subsequent experiments. The responses to microinjections of NMDA into the ARCN were completely blocked by prior microinjections of D-AP7 (10 mM) into the same ARCN (n = 4). In this and other series of experiments, all microinjections were unilateral unless mentioned otherwise.

**Table 1:** Concentration-response for microinjections of NMDA into the ARCN.

NMDA concentration (mM)	n	Decrease in MAP (mmHg)	Increase in HR (bpm)
2.5	4	5.3±1.4‡	8.5±0.6†
5	4	10.3±1.7**	22.3±5.1
10	4	11.8±1.7**	25.0±7.2*

ARCN: the hypothalamic arcuate nucleus; bpm: beats/min; HR: heart rate; MAP: mean arterial pressure; NMDA: N-methyl-D-aspartic acid. **Significantly greater compared with ‘‡’ (P<0.01). *Significantly greater compared with ‘†’ (P<0.05). The volume of all microinjections was 20 nl.

### 2. NMDA-induced Responses in Different Parts of the ARCN

In order to compare cardiovascular responses elicited from different parts of the ARCN, this nucleus was arbitrarily divided into rostral, middle and caudal regions. Cardiovascular responses to microinjections of NMDA in these three regions are shown in Table 2. The decreases in MAP and increases in HR elicited from these three regions of ARCN were not significantly different.

**Table 2:** Cardiovascular responses to microinjections of NMDA in different ARCN regions.

Region	n	Coordinates (mm caudal to bregma)	Decrease in MAP (mmHg)	Increase in HR (bpm)
Rostral	5	1.9–2.6	12.2±4.1	22.8±2.5
Middle	5	2.7–3.4	10.8±2.4	25.2±5.0
Caudal	5	3.5–4.1	12.4±2.1	25.0±6.1

ARCN: the hypothalamic arcuate nucleus; bpm: beats/min; HR: heart rate; MAP: mean arterial pressure; NMDA: N-methyl-D-aspartic acid (10 mM, 20 nl). There was no significant difference in the cardiovascular responses elicited in the rostral, middle and caudal regions of ARCN.

### 3. Reproducibility of NMDA Responses Elicited from the ARCN

NMDA was microinjected into the caudal ARCN (3.5–4.1 mm caudal to the bregma) 3 times at 20-min intervals (n = 4). The decreases in MAP in response to the 3 consecutive microinjections of NMDA were 11.5±1.3, 11.3±1.2 and 11.8±0.9 mmHg, respectively (P>0.05). The increases in HR were 22.0±4.8, 21.8±3.1 and 24.3±2.5 bpm, respectively (P>0.05). Thus, no tachyphylaxis was observed with repeated microinjections of NMDA into the ARCN when the interval between the injections was at least 20 min.

### 4. Site Specificity of the Responses to the Microinjections of NMDA

In order to investigate whether the cardiovascular responses elicited from the ARCN were site-specific, NMDA (10 mM, 20 nl) was microinjected into other adjacent regions. Microinjections of NMDA into the DMN elicited pressor (11.8±4.6 mmHg) and tachycardic (25.8±7.8 bpm) responses (n = 4). Microinjections of NMDA into the third ventricle also elicited pressor (13.8±5.7 mmHg) and tachycardic (35.1±6.8 bpm) responses (n = 4). Microinjections of NMDA into the VMN, however, elicited depressor (10.8±3.7 mmHg) and tachycardic (21.1±7.1 bpm) responses (n = 4).

### 5. ARCN Stimulation: Effect of Fasting on Cardiovascular Responses

In order to examine whether cardiovascular responses to microinjections of NMDA into the ARCN were affected by feeding, experiments were done in fasted rats. Rats were fasted for 24 hrs while drinking water was allowed ad libitum. In this group of rats (n = 4), baseline MAP (98.5±2.9 mmHg) and HR (439.8±6.0 bpm) were not different from those in non-fasted rats (P>0.05). Microinjections of NMDA into the ARCN in fasted rats elicited decreases in MAP (13.3±2.3 mmHg) and increases in HR (29.5±5.7 bpm); these responses were not statistically different from those elicited in non-fasted rats (P>0.05).

### 6. ARCN Stimulation: Effect of GABA-A Receptor Blockade in the PVN

**Figure 1:**
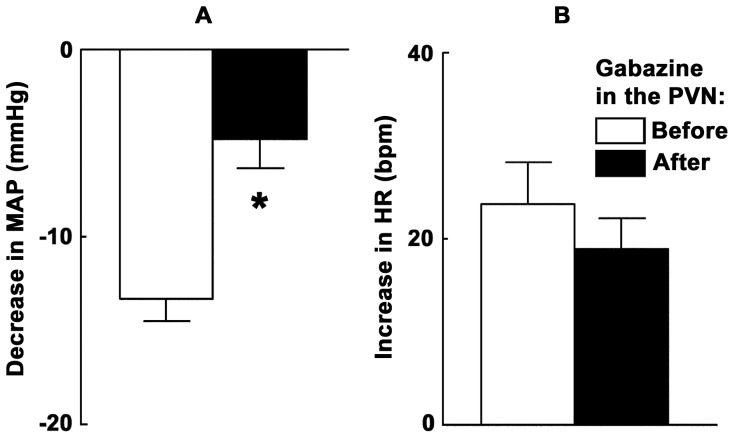
ARCN stimulation: effect of GABA-A receptor blockade in the PVN. Bar graphs (n = 6) showing significant (*P<0.01) attenuation of depressor responses (A), but not tachycardic responses (B), elicited by microinjections of NMDA into the ARCN following the blockade of GABA-A receptors in the ipsilateral PVN. In this and other figure legends: ARCN, the hypothalamic arcuate nucleus; GABA, gamma-aminobutyric acid; HR, heart rate (beats/min); MAP, mean arterial pressure (mmHg); NMDA, N-methyl-D-aspartic acid; PVN, the hypothalamic paraventricular nucleus; Gabazine, GABA-A receptor antagonist.

Microinjection of gabazine (1 mM) into the PVN attenuated the depressor response, but not tachycardic response, to microinjection of NMDA into the ipsilateral ARCN. Group data (n = 6) for this experiment are shown in Figure 1. In these experiments, NMDA was used to stimulate the middle or caudal ARCN and 20 min later gabazine (1 mM) was microinjected into the ipsilateral PVN; this concentration of gabazine was selected based on our previous reports [38]. Microinjections of gabazine into the PVN increased the baseline MAP (11.2±2.2 mmHg) and HR (44.5±10.4 bpm); similar effects of GABA receptor blockade in the PVN have been reported previously [19]. After an interval of 15–20 min, when there was 80% recovery of the MAP, microinjection of NMDA into the ARCN was repeated. The decreases in MAP in response to NMDA in the ARCN before and after the microinjections of gabazine into the PVN were 13.3±1.2 and 4.8±1.5 mmHg, respectively (P<0.01, Figure 1A) and the increases in HR were 23.7±4.5 and 19.0±3.2 bpm, respectively (P>0.05, Figure 1B). The cardiovascular responses elicited by the microinjections of NMDA into the ARCN were not significantly altered by microinjections of either aCSF or distilled water (pH 7.4) into the PVN (n = 4, each group; data not shown).

In another group of rats (n = 4), microinjections of gabazine (1 mM) into the PVN significantly exaggerated MAP responses to the microinjections of NMDA into the same PVN site. The increases in MAP in response to the microinjections of NMDA in the PVN before and after the microinjections of gabazine into the PVN were 15.2±4.1 and 26.0±3.4 mmHg, respectively (P<0.05). On the other hand, the microinjections of gabazine into the PVN did not significantly alter tachycardic responses to NMDA; the increases in HR elicited by NMDA before and after the microinjections of gabazine were 34.4±8.9 and 28.8±4.8 bpm, respectively (P>0.05**)**.

### 7. ARCN Stimulation: Effect of Gabazine Microinjections into the Same Nucleus

**Figure 2:**
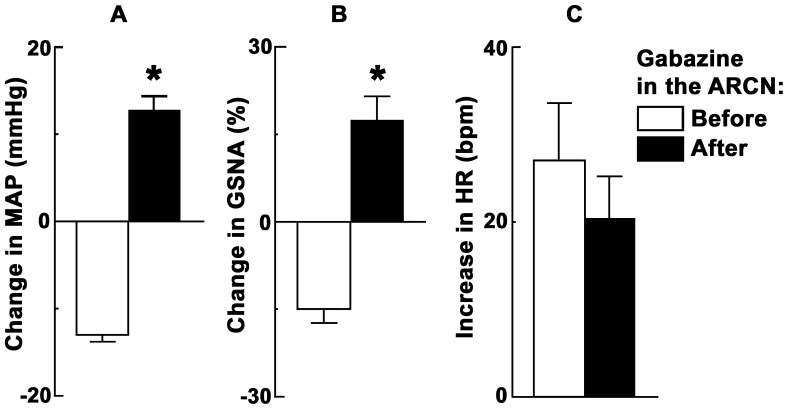
ARCN stimulation: effect of gabazine microinjections into the same nucleus. Bar graphs (n = 5) showing conversion of decreases in MAP and GSNA elicited by microinjections of NMDA into the ARCN to increases in MAP and GSNA (*P<0.01) following the blockade of GABA-A receptors in the same nucleus (A and B, respectively). Tachycardic responses elicited by the microinjections of NMDA into the ARCN were not altered significantly by the blockade of GABA-A receptors in the same nucleus (C). GSNA: greater splanchnic nerve activity.

In these experiments, NMDA was first microinjected into the ARCN; usual decreases in MAP (13.0±0.8 mmHg) and GSNA (15.0±2.4%) and increases in HR (27.0±6.6 bpm) were elicited (n = 5) (Figure 2). Twenty min later, microinjections of gabazine (1 mM) alone into the ARCN increased the baseline MAP (10.8±2.0 mmHg), GSNA (17.2±4.3%) and HR (31.3±5.2 bpm). After an interval of 15–20 min, when there was 80% recovery of the gabazine-induced increases in baseline MAP, GSNA and HR, NMDA microinjections were repeated at the same ARCN site; increases (instead of decreases) in MAP (12.8±1.5 mmHg, (P<0.01; Figure 2A) and GSNA (17.4±4.1%, (P<0.01; Figure 2B) were elicited. Increases in HR elicited by NMDA after the microinjections of gabazine (20.4±4.8 bpm) were not statistically different (P>0.05; Figure 2C).

### 8. ARCN Stimulation: Effect of NPY Receptor Blockade in the PVN

**Figure 3:**
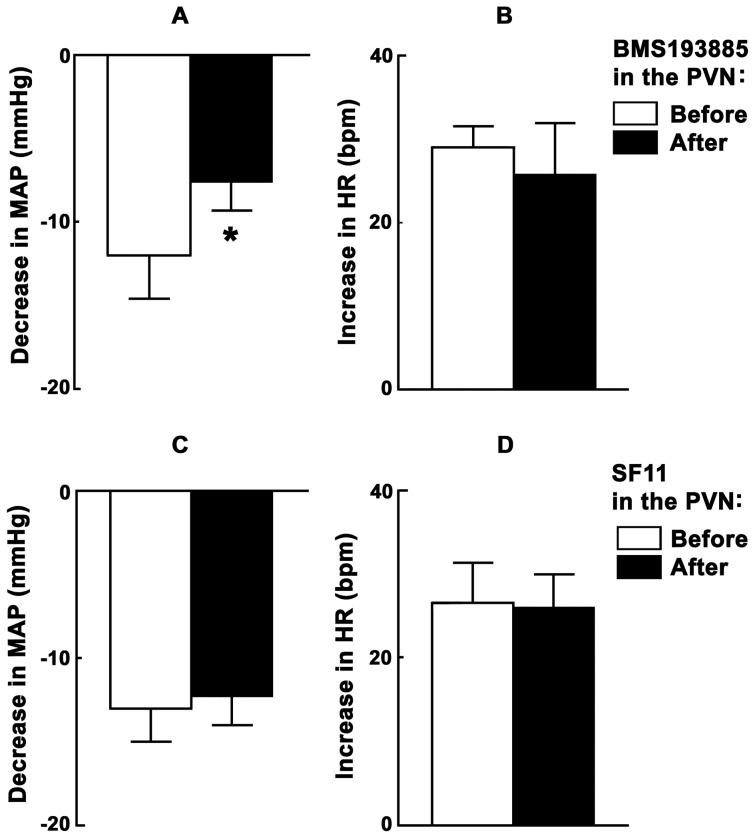
ARCN stimulation: effect of NPY receptor blockade in the PVN. Bar graphs (n = 5) showing significant (*P<0.05) attenuation of depressor responses (A), but not tachycardic responses (B), elicited by microinjections of NMDA into the ARCN following the blockade of NPY1 receptors in the ipsilateral PVN. The blockade of NPY2 receptors in the ipsilateral PVN (n = 5) did not significantly alter the depressor (C) and tachycardic (D) responses elicited by microinjections of NMDA into the ARCN. NPY, neuropeptide Y; BMS193885, NPY1 receptor antagonist; SF11, NPY2 receptor antagonist.

Microinjections of NMDA were used to stimulate the middle or caudal ARCN and BMS193885 (NPY1 receptor antagonist; 15 mM) was microinjected 20 min later into the ipsilateral PVN. NMDA microinjection was repeated into the ARCN 2–3 min later. Group data (n = 5) for this experiment are shown in Figure 3. Microinjections of BMS193885 into the PVN attenuated the depressor responses elicited by the microinjections of NMDA into the ARCN; the decreases in MAP before and after the microinjections of BMS193885 were 12.0±2.6 and 7.6±1.7 mmHg, respectively (P<0.05, Figure 3A). The tachycardic responses to the microinjections of NMDA into the ARCN were not altered by the microinjections of BMS193885 into the PVN; the increases in HR in response to NMDA in the ARCN before and after the microinjections of BMS193885 into the PVN were 29.0±2.5 and 25.8±6.0 bpm, respectively (P>0.05, Figure 3B). Microinjections of a smaller concentration of BMS193885 (10 mM) into the PVN did not alter any cardiovascular responses to the microinjection of the NMDA into the ARCN (n = 4; data not shown).

In another group of rats (n = 5), SF11 (NPY2 receptor antagonist; 5 mM) microinjected into the PVN did not alter the cardiovascular responses to the microinjections of NMDA into the ipsilateral ARCN. The decreases in MAP in response to NMDA in the ARCN before and after the microinjections of SF11 into the PVN were 13.0±2.0 and 12.3±1.7 mmHg, respectively (P>0.05, Figure 3C). Similarly, the increases in HR were 26.5±4.8 and 26.0±4.0 bpm, respectively (P>0.05, Figure 3D). Microinjections of either BMS193885 (15 mM) or SF11 (5 mM) alone into the PVN did not elicit significant changes in MAP or HR and did not alter any cardiovascular responses to the microinjections of the NMDA at the same site (n = 4; data not shown).

Most of NPY containing neurons in the ARCN co-express AgRP [2]. Therefore, it is possible that AgRP may also be released in the PVN following the ARCN stimulation. Cardiovascular responses to microinjections of AgRP into the PVN were, therefore, tested. Microinjections of AgRP (1 mM) into the PVN elicited no BP or HR responses (n = 4). The concentration of AgRP (1 mM) used in these experiments, was selected from published literature [39].

### 9. ARCN Stimulation: Effect of NPY Receptor Blockade in the Same Nucleus

In another experiment (n = 4), microinjections of BMS193885 (15 mM) into the ARCN did not alter any cardiovascular responses to the microinjections of the NMDA into the same site of ARCN. The decreases in MAP elicited by the microinjections of NMDA into the ARCN before and after microinjections of BMS193885 into the same ARCN site were 13.5±2.5 and 13.0±1.5 mmHg, respectively (P>0.05). Similarly, the increases in HR were 29.8±7.0 and 24.3±3.6 bpm, respectively (P>0.05).

### 10. ARCN Stimulation: Effect of Opioid Receptor Blockade in the PVN

**Figure 4:**
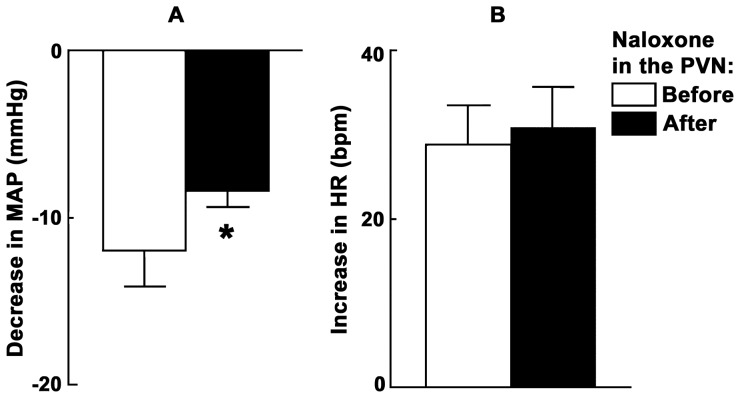
ARCN stimulation: effect of opioid receptor blockade in the PVN. Bar graphs (n = 5) showing significant (*P<0.05) attenuation of depressor responses (A), but not tachycardic responses (B), elicited by microinjections of NMDA into the ARCN following the blockade of opioid receptors in the ipsilateral PVN. Naloxone: opioid receptor antagonist.

Microinjections of NMDA were used to stimulate the middle or caudal ARCN and naloxone (20 mM) was microinjected 20 min later into the ipsilateral PVN. NMDA microinjection was repeated into the ARCN 2–3 min later. Group data for this experiment are shown in Figure 4. In this group of rats (n = 5), depressor responses to the microinjections of NMDA into the ARCN were significantly attenuated by naloxone microinjected into the ipsilateral PVN. The decreases in MAP in response to NMDA in the ARCN before and after the microinjections of naloxone into the PVN were 12.0±2.1 and 8.4±1.0 mmHg, respectively (P<0.05; Figure 4A). Tachycardic responses to microinjections of NMDA in the ARCN, however, were not altered by naloxone. The increases in HR in response to NMDA in the ARCN before and after the microinjections of naloxone into the PVN were 28.8±4.7 and 31.0±4.6 bpm, respectively (P>0.05; Figure 4B). This concentration of naloxone is smaller than the concentrations used by others [40]. The microinjection of naloxone alone into the PVN elicited slight increases in MAP (3.0±1.5 mmHg) and HR (5.6±4.6 bpm). The microinjections of naloxone (20 mM) into the PVN did not alter any cardiovascular responses to the microinjections of the NMDA into the same site of PVN (n = 4; data not shown).

### 11. ARCN Stimulation: Effect of Opioid Receptor Blockade in the Same Nucleus

In another experiment (n = 4), microinjections of naloxone (20 mM) into the ARCN did not alter any cardiovascular responses to microinjections of the NMDA into the same site of ARCN. The decreases in MAP elicited by the microinjections of NMDA into the ARCN before and after the microinjections of naloxone into the same site were 11.3±1.0 and 11.8±0.9 mmHg, respectively (P>0.05). Similarly, the increases in HR were 26.8±4.8 and 23.5±5.5 bpm, respectively (P>0.05).

### 12. ARCN Stimulation: Effect of Combined GABA-A, NPY1 and Opioid Receptor Blockade in the PVN

**Figure 5:**
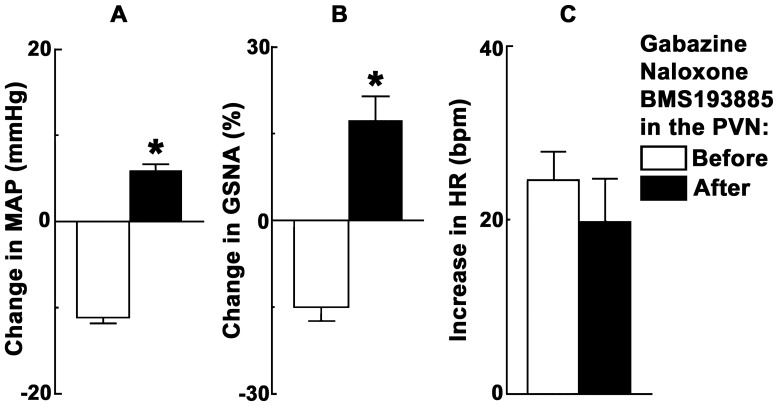
ARCN stimulation: data showing effect of GABA-A, NPY1 and opioid receptor blockade in the PVN. Bar graphs (n = 6) showing conversion of decreases in MAP and GSNA elicited by microinjections of NMDA into the ARCN to increases in MAP and GSNA (*P<0.01) following the combined blockade of GABA-A, NPY1 and opioid receptors in the ipsilateral PVN (A and B, respectively). Tachycardic responses elicited by the microinjections of NMDA into the ARCN were not altered significantly by the combined blockade of these receptors in the PVN (C).

**Figure 6:**
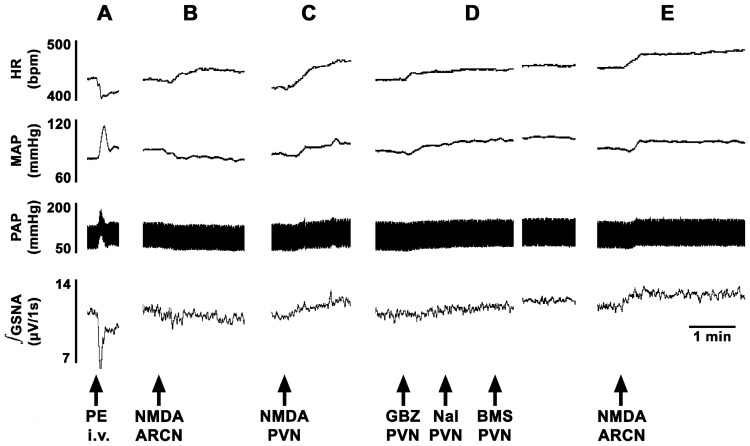
ARCN stimulation: tracing showing effect of GABA-A, NPY1 and opioid receptor blockade in the PVN. Top trace: HR (beats/min), second trace: MAP (mmHg), third trace: PAP (mmHg), bottom trace: integrated GSNA (µV/1s). A: bolus injection of PE (10 µg/kg, i.v.) elicited increase in MAP, reflex bradycardia and reflex inhibition of GSNA. B: Unilateral microinjection of NMDA (10 mM, 20 nl) into the ARCN elicited decreases in MAP and GSNA and an increase in HR. C: 20 min later, ipsilateral PVN site was identified by a microinjection of NMDA (10 mM, 30 nl); increases in MAP, HR and GSNA were elicited. D: 20 min later, gabazine (1 mM), naloxone (20 mM) and BMS193885 (15 mM) (30 nl each) were microinjected into the ipsilateral PVN; increases in MAP, HR and GSNA were elicited (a gap in the tracings was inserted to accommodate the peak response). E: After an interval of 15 min, when the MAP showed about 80% recovery, NMDA (10 mM, 20 nl) was again microinjected into the ARCN; decreases in MAP and GSNA were converted to increases in MAP and GSNA but tachycardic responses remained unchanged. BMS, BMS193885; GBZ, gabazine; GSNA, greater splanchnic nerve activity; Nal, naloxone; PAP, pulsatile arterial pressure; PE, phenylephrine.

In another group of rats (n = 6), combined blockade of GABA-A, NPY1 and opioid receptors in the PVN was accomplished by microinjections (30 nl each) of gabazine (1 mM), BMS193885 (15 mM), and naloxone (20 mM), respectively; an increase in the baseline MAP (10.9±3.4 mmHg), HR (35.3±9.0 bpm) and GSNA (19.9±3.3%) was observed. The baseline MAP showed 80% recovery within 15 min. At this time, the decreases in MAP and GSNA elicited by the microinjections of NMDA into the ipsilateral ARCN were converted to increases in MAP and GSNA. However, tachycardic responses to ARCN stimulation with NMDA were not affected. The changes in MAP elicited by the microinjections of NMDA into the ARCN before and after the microinjections of these three antagonists into the PVN were −11.1±0.7 and +5.9±0.7 mmHg, respectively (P<0.01, Figure 5A). Similarly, the changes in GSNA were −15.0±2.4 and +17.4±4.1%, respectively (P<0.01, Figure 5B). The increases in HR were 24.5±3.3 and 19.9±4.8 bpm, respectively (P>0.05, Figure 5C). Typical tracings of this experiment are shown in Figure 6.

### 13. Effect of Baseline MAP on Cardiovascular Responses Elicited from the ARCN

The following experiment was done to investigate the role of baseline MAP in determining the type of cardiovascular response elicited from the ARCN. When baseline MAP was 100.3±4.9 mmHg, depressor responses were elicited by microinjection of NMDA into the ARCN (n = 4). The MAP was then lowered by an infusion of SNP (150–300 µg/kg/hour), using an infusion pump (Sage Instruments, model 341). When the baseline MAP was steady at 73.5±2.2 mmHg, microinjection of NMDA into the ARCN elicited pressor responses. Chemical stimulation of the ARCN always elicited tachycardia. These data are shown in Table 3.

**Table 3:** Effect of baseline MAP on cardiovascular responses elicited by microinjections of NMDA into the ARCN.

	SNP infusion (n = 4)	
	Before	After
Baseline MAP (mmHg)	100.3±4.9†	73.5±2.2*
Change in MAP (mmHg)	−12.5±1.4‡	+15.0±1.5**
Baseline HR (bpm)	429.5±15.8§	452.8±10.3***
Increase in HR (bpm)	25.5±4.6	28.5±5.5

* Significantly smaller compared with ‘†’ (P<0.01). **Significantly greater compared with ‘‡’ (P<0.01). ***Significantly greater compared with ‘§’ (P<0.05). ARCN, the hypothalamic arcuate nucleus; HR, heart rate (beats/min); MAP, mean arterial pressure, NMDA, N-methyl-D-aspartic acid (10 mM, 20 nl); SNP, sodium nitroprusside (150–300 µg/kg/hr).

### 14. ARCN Stimulation: Effect of MC Receptor Blockade in the PVN

In these experiments (n = 5), NMDA was microinjected into the ARCN and SHU9119 (MC3/4 receptor antagonist; 2 mM) was microinjected into the ipsilateral PVN 20 min later. The concentration of SHU9119 used was selected from published literature [41]. Microinjections of SHU9119 alone into the PVN did not elicit significant changes in the baseline MAP or HR. NMDA microinjection was repeated into the ARCN 2–3 min later. The decreases in MAP in response to NMDA in the ARCN before (10.6±0.9 mmHg) and after (11.6±1.1 mmHg) the microinjections of SHU9119 into the PVN were not statistically different (P>0.05). Similarly, the increases in HR before (26.2±2.8 bpm) and after (26.8±2.7 bpm) the microinjections of SHU9119 into the PVN were not statistically different (P>0.05). In the same group of rats, the microinjections of SHU9119 did not alter any cardiovascular responses to the microinjection of the NMDA at the same site (i.e., the PVN) (data not shown).

In another group of rats (n = 5), the baseline BP was lowered by intravenous infusion of SNP; the baseline BP and HR during SNP infusion were 75.6±0.7 mmHg and 451.8±8.7 bpm, respectively. As mentioned in section 13, at baseline BP below normal, pressor and tachycardic responses were elicited by microinjections of NMDA into the ARCN. These responses were significantly attenuated by microinjections of SHU9119 into the ipsilateral PVN. The increases in MAP in response to NMDA in the ARCN before and after microinjections of SHU9119 into the ipsilateral PVN were 14.6±1.7 and 10.2±2.1 mmHg, respectively (P<0.01). Similarly, the increases in HR before and after microinjections of SHU9119 into the ipsilateral PVN were 27.6±4.9 and 18.4±2.6 bpm, respectively (P<0.05).

### 15. Identification of GAD67, NPY and Beta-endorphin Immunoreactive ARCN Cells Retrogradely Labeled from the PVN

**Figure 7:**
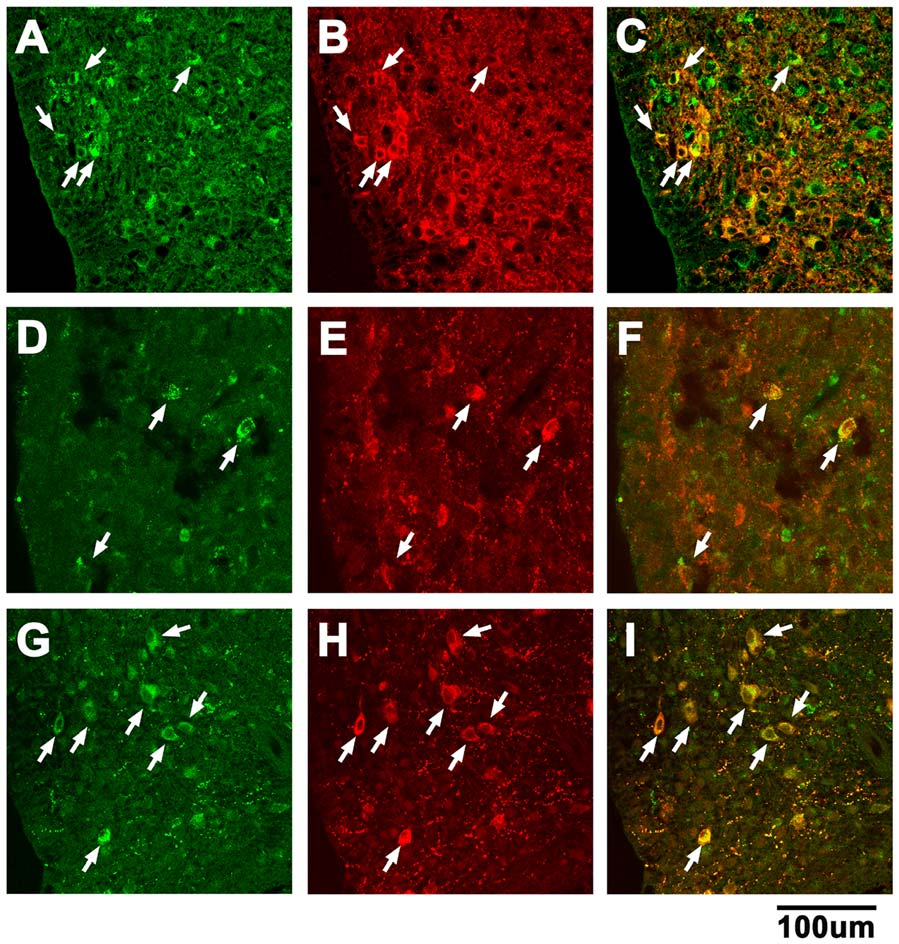
Identification of GAD67, NPY and beta-endorphin immunoreactive ARCN cells retrogradely labeled from the PVN. A: Retrogradely labeled neurons in the ARCN after unilateral microinjection (30 nl) of green retrobeads IX into the ipsilateral PVN. B: GAD67 immunoreactive neurons in the section shown in A. C: The merged images of A and B indicated that some retrogradely labeled cells in the ARCN contained GAD67 (white arrows). D: ARCN neurons retrogradely labeled from PVN (different rat). E: NPY immunoreactive neurons in the section shown in D. F: The merged images of D and E indicated that some retrogradely labeled cells in the ARCN contained NPY (white arrows). G: ARCN neurons retrogradely labeled from PVN (different rat). H: Beta-endorphin immunoreactive neurons in the section shown in G. I: The merged images of G and H indicated that some retrogradely labeled cells in the ARCN contained beta-endorphin (white arrows). GAD67: glutamic acid decarboxylase 67.

Unilateral microinjection of green retrobeads IX into the PVN resulted in retrograde labeling of ARCN neurons with ipsilateral preponderance (Figure 7, A, D and G). GAD67, NPY and beta-endorphin immunoreactive neurons in the same sections (i.e., Figure 7, A, D and G) are shown in Figure 7, B, E and H, respectively. The merged images indicated that some retrogradely labeled cells in the ARCN contained GAD67 (Figure 7C), NPY (Figure 7F) and beta-endorphin (Figure 7I).

### 16. Histological Identification of Microinjection Sites

**Figure 8:**
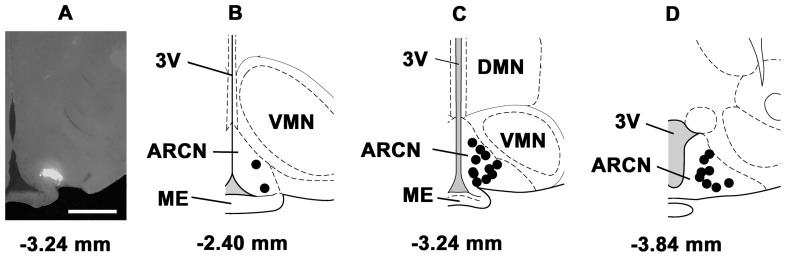
Histological identification of microinjection sites in the ARCN. A: A typical microinjection site in the ARCN marked with green retrobeads IX (20 nl). B–D: Composite diagrams of ARCN sections at levels 2.40 mm (rostral region), 3.24 mm (middle region) and 3.84 mm (caudal region) showing microinjection sites (n = 21 rats); the coordinates mentioned below each drawing are caudal to the bregma. Each dark spot represents one microinjection site in one animal. Bar in panel A = 500 µm. DMN: the hypothalamic dorsomedial nucleus; ME: the hypothalamic median eminence; VMN: the hypothalamic ventromedial nucleus; 3V: the third ventricle.

**Figure 9:**
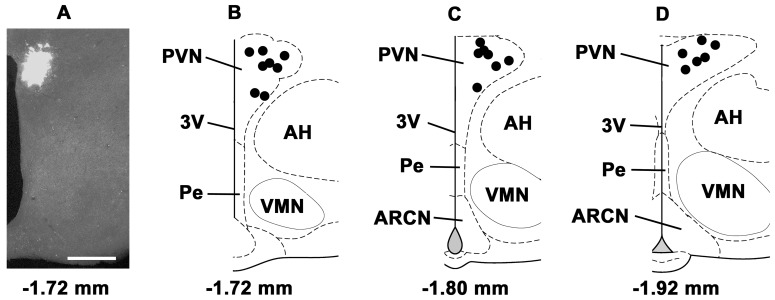
Histological identification of microinjection sites in the PVN. A: A typical microinjection site in the PVN marked with green retrobeads IX (30 nl). B–D: Composite diagrams of PVN sections at levels 1.72 mm, 1.80 mm, 1.92 mm caudal to the bregma showing microinjection sites (n = 21 rats). Each dark spot represents one microinjection site in one animal. Bar in panel A = 500 µm. AH: the hypothalamic anterior nucleus; Pe: the hypothalamic periventricular nucleus.

The microinjections sites in the ARCN were marked with diluted green retrobeads IX (n = 21 rats). A typical ARCN site and composite diagrams of the ARCN microinjection sites are shown in Figure 8. Similarly, the PVN microinjection sites were marked in 21 rats. A typical microinjection site in the PVN and composite diagrams are presented in Figure 9. The volumes of green retrobeads IX microinjected into the ARCN and the PVN were 20 and 30 nl, respectively.

## Discussion

The following new observations were made in this study: 1) at normal baseline MAP, unilateral chemical stimulation of the ARCN by microinjections of NMDA induced depressor and tachycardic responses in anesthetized rats, 2) at below normal baseline MAP, microinjections of NMDA into the ARCN elicited pressor and tachycardic responses, 3) the depressor responses were mediated via GABA-A, NPY1 and opioid receptors in the ipsilateral PVN, 4) combined blockade of GABA-A, NPY1 and opioid receptors in the ipsilateral PVN converted decreases in BP and GSNA to increases in BP and GSNA, 5) the decreases in MAP and GSNA elicited by the chemical stimulation of the ARCN at normal baseline MAP were converted to increases in MAP and GSNA after the blockade of GABA-A receptors, but not opioid or NPY1 receptors, in the ARCN, and 6) the pressor and tachycardic responses elicited by the stimulation of ARCN at below normal baseline MAP were attenuated by the blockade of MC3/4 receptors in the ipsilateral PVN.

Generally higher concentrations of NMDA and L-Glu were needed to elicit responses from the ARCN. The reason for the need to use higher concentrations of NMDA and L-Glu to stimulate the ARCN, when compared to the concentrations needed for stimulation of medullary cardiovascular areas, may be that the ARCN neurons are tonically inhibited by GABA [2]. However, the concentrations of NMDA used (10 mM) did not exert non-specific effects because D-AP7 (an NMDA receptor antagonist; 10 mM, 20 nl) completely blocked the effects of NMDA.

As mentioned in Introduction section, there are numerous reports on the role of the ARCN in feeding behavior and energy homeostasis [1]–[5]. Nutrients as well circulating hormones (e.g., ghrelin, leptin and insulin) can affect the levels of peptides in the NPY/AgRP and POMC/CART neurons in the ARCN. In order to avoid the variability of responses due to differences in the peptide levels in the ARCN caused by the differences in food intake of different rats, experiments were done on fasted rats. The baseline MAP and HR were not different in fasted rats compared to the fed rats. NMDA microinjections into the ARCN elicited depressor and tachycardic responses in fasted rats; these responses were not statistically different from those in the fed rats.

The presence of GABA, NPY, and beta-endorphin containing neurons has been reported in the ARCN as mentioned in the Introduction section [8], [9], [11]–[14]. The blockade of GABA-A receptors by gabazine or opioid receptors by naloxone or NPY1 receptors by BMS193885 in the PVN attenuated the depressor responses to the chemical stimulation of the ipsilateral ARCN. Gabazine, naloxone and BMS193885 have been reported to be selective antagonists for GABA-A, opioid and NPY1 receptors [38], [42], [43]. The concentrations of gabazine, naloxone and BMS193885 that were used to block their respective receptors in the PVN did not alter the responses to microinjections of NMDA at the same site suggesting that these antagonists did not exert any deleterious effects at the microinjection site. Based on these observations, it was concluded that activation of GABA, beta-endorphin and NPY containing neurons in the ARCN resulted in the release of these neurotransmitters in the ipsilateral PVN. GABA, beta-endorphin and NPY have been reported to have inhibitory effects on PVN neurons [30]–[32]. It is well established that disinhibition or chemical stimulation of the PVN excites spinally projecting RVLM neurons [19]–[21], [23]–[26]. Therefore, inhibition of PVN neurons is expected to result in decrease in MAP and GSNA as observed in this study.

Combined blockade of GABA-A, NPY1 and opioid receptors in the PVN converted decreases in BP and SNA in response to ARCN stimulation into increases in MAP and SNA. These results can be explained as follows. The presence of POMC/CART neurons has been reported in the ARCN and these neurons are known to project to the PVN [1]–[5]. Stimulation of these neurons in the ARCN by microinjections of NMDA is expected to release alpha-MSH, one of the cleavage products of POMC [10], [44], into the PVN. Alpha-MSH has been reported to cause depolarization of PVN neurons and increase their firing activity [45]. The release of CART, if any, in the PVN is also expected to stimulate PVN neurons based on the information that it has stimulatory effects on neurons [46]. Stimulation of PVN neurons has been reported to increase MAP and SNA [17].

When the baseline MAP was lowered to below normal levels by an infusion of SNP, pressor responses (instead of depressor response) were elicited by the stimulation of ARCN. In this experiment, activation of POMC neurons in the ARCN by NMDA may have resulted in the release of alpha-MSH in the PVN and elicit a pressor response. The effects of alpha-MSH are mediated via MC3/4 receptors. Therefore, blockade of MC3/4 receptors in the PVN by SHU9119 attenuated the excitatory effects of the ARCN on PVN presympathetic neurons and thus attenuated the pressor responses elicited by ipsilateral ARCN stimulation. Similar mechanism may be responsible for the attenuation of the tachycardic responses elicited by ARCN stimulation because HR responses elicited by ARCN stimulation are partly mediated via the sympathetic input to the heart [29]. However, increases in HR elicited by stimulation of the ARCN are also mediated via inhibition of vagal input to the heart [29]. The pathways in the brain that mediate decrease in vagal input to the heart have not been delineated yet and the effect of SHU9119 on these pathways is not known.

We have previously reported [28], [29] that microinjections of NMDA into the ARCN elicited increases in MAP, HR and GSNA. In this study, we report that microinjections of NMDA into the ARCN elicited decreases in MAP, HR and GSNA. Based on these observations, we have noticed that the type of cardiovascular responses elicited by the stimulation of the ARCN depended on baseline MAP. When the baseline MAP was relatively lower (e.g., 86.9±1.5 mmHg) [28], pressor responses were elicited from the ARCN while at relatively higher baseline MAP (e.g., 99.7±1.1 mmHg; this study), depressor responses were elicited by ARCN stimulation. Indeed, in this study, when the baseline MAP was decreased by an intravenous infusion of SNP, depressor responses to the ARCN stimulation were converted into pressor response. These changes in the type of responses elicited by the stimulation of ARCN can be explained as follows. As mentioned in the Introduction section, the ARCN includes POMC/CART and NPY/AgRP neurons. POMC/CART neurons contain one of excitatory neurotransmitters (alpha-MSH). NPY/AgRP neurons contain inhibitory neurotransmitters (e.g., NPY, GABA and opiates) [4], [5]. Microinjections of NMDA into the ARCN are expected to stimulate both POMC/CART and NPY/AgRP neurons. Both types of ARCN neurons are known to project to the PVN [4], [5]. Therefore, both excitatory (e.g., alpha-MSH) and inhibitory (e.g., NPY, GABA and opiates) neurotransmitters are released in the PVN. However, the effect of inhibitory neurotransmitters prevails at normal baseline MAP because the target neurons in the RVLM are less excitable due to tonic inhibitory effect of baroreceptor afferents. In this context, it may be recalled that activation of baroreceptor afferents stimulates NTS neurons, these neurons excite GABAergic CVLM neurons which, in turn, inhibit RVLM neurons [47], [48]. When this tonic inhibitory influence on RVLM neurons is reduced by lowering baseline MAP (e.g., by SNP infusion), the effects of excitatory inputs from the PVN to the RVLM are unmasked and pressor responses are elicited. The excitatory inputs in the PVN are activated by the release of excitatory neurotransmitters (e.g., alpha-MSH) following the stimulation of the ARCN. Indeed, the pressor responses were attenuated by microinjections of SHU9119 (MC3/4 receptor antagonist) in the PVN in this study.

The blockade of GABA-A, NPY1 and opioid receptors in the PVN did not alter the tachycardic responses elicited by microinjections of NMDA into the ARCN. We have previously reported that the increases in HR elicited from the ARCN are mediated via the activation of spinal cord ionotropic glutamate receptors (iGLURs) and decreases in vagal input to the heart [29]. Vagal input to the heart is more dominant in regulating HR [49]. Our results are consistent with this report because tachycardic responses were elicited by microinjections of NMDA into the ARCN despite the concomitant decrease in GSNA.

When gabazine was microinjected into the ARCN instead of PVN, it converted depressor responses to NMDA microinjections into the ARCN to pressor responses. This observation suggested that GABA-containing neurons in the ARCN (e.g., NPY/AgRP neurons) [1], [4], may tonically inhibit the POMC/CART neurons which contain excitatory neurotransmitters such as alpha-MSH [11]–[14].

Microinjections of gabazine into the PVN exaggerated the pressor responses elicited by microinjections of NMDA into the same nucleus. This effect can be explained as follows. The PVN neurons are under tonic inhibition from GABAergic inputs [50]. Therefore, blockade of these inhibitory inputs by gabazine may render the PVN neurons more excitable and elicit exaggerated responses to NMDA. On the other hand, microinjections of BMS193885 (NPY1 receptor antagonist) or naloxone (opioid receptor antagonist) did not alter the response to NMDA in the PVN.

In our experiments where NMDA was microinjected into the ARCN and antagonists for GABA-A, NPY1 and opioid receptors were microinjected into the ipsilateral PVN, it may be argued that antagonists microinjected into the PVN may have diffused into the ipsilateral ARCN and altered the responses elicited from this nucleus. In these experiments, NMDA was microinjected into either middle or caudal regions of the ARCN. The distance between these regions of the ARCN and ipsilateral PVN is 1.8–3.3 mm. The volumes of microinjections into the ARCN and PVN were 20 and 30 nl, respectively. The diameter of the diffusion sphere of these microinjection volumes has been estimated to be less than 576 µm [51] which makes it unlikely that antagonists injected into the PVN diffused to the ARCN or vice versa. Furthermore, when BMS193885 or naloxone was microinjected into the PVN, the depressor responses to microinjections of NMDA into the ARCN were attenuated. On the other hand, when these antagonists were microinjected directly into the ARCN, they did not alter the depressor responses elicited by microinjections of NMDA into the same nucleus indicating that microinjections of these antagonists into the PVN did not spread to the ARCN.

Although microinjections of NMDA into the VMN elicited responses similar to those of ARCN (depressor and tachycardic responses), it is unlikely that the cardiovascular responses elicited from the ARCN were mediated via the VMN because the responses elicited from the ARCN persisted even in the region where VMN is not present (e.g., 3.5–4.1 mm caudal to the bregma) [37]. Diffusion of NMDA microinjected into the ARCN to adjacent the DMN or third ventricle was also ruled out because pressor, instead of depressor, responses were elicited from these structures.

Our retrograde tracing combined with immunohistochemical studies demonstrated the presence GAD67, NPY, and beta-endorphin immunoreactive neurons that projected to the ipsilateral PVN. These data are in agreement with earlier reports in which the presence of such neurons has been demonstrated in the ARCN [1]–[5], [8], [9], [52]. Both NPY/AgRP and POMC/CART neurons have been reported to contain GABA [1], [4]. Thus, our GAD67 immunoreactive neurons may be either NPY/AgRP neurons or POMC/CART neurons. Beta-endorphin has been reported to be one of the products present in POMC neurons [10]. Thus, our beta-endorphin immunoreactive neurons may be POMC/CART neurons. The results of our immunohistochemical studies were consistent with our conclusion that activation of GABA, NPY and beta-endorphin containing neurons in the ARCN elicited depressor responses via activation of GABA-A, NPY1 and opioid receptors, respectively, in the ipsilateral PVN. NPY and GABA may be co-released in the PVN by microinjections of NMDA into the ARCN.

### Conclusion and Significance

The ARCN is located in close proximity to the median eminence which has a high capillary density (1079/mm^2^), high blood flow (2433 µl/g/min) and lacks blood-brain barrier [53]. It has been suggested that this anatomic arrangement of the ARCN may favor the accessibility of the ARCN neurons to physiologically active substances circulating in the blood [54]. For example, circulating leptin and insulin have been reported to influence the activity of ARCN neurons [4], [15], [16]. Obesity, which is a risk factor for cardiovascular diseases, is a growing health problem today. The role of ARCN in the regulation of feeding behavior and energy expenditure has been extensively studied [1]–[5] but little information is available regarding its role in cardiovascular regulation. The results of the present study provide a platform on which the future studies can be designed to explore the role of ARCN in obesity-related cardiovascular disease.
